# P-1820. Can a Tele-Mentoring Program for Antimicrobial Stewardship Contribute to Program Satisfaction and Staff Retention?

**DOI:** 10.1093/ofid/ofae631.1983

**Published:** 2025-01-29

**Authors:** Zahra Kassamali-Escobar, Jeannie D Chan, Whitney Hartlage, Maria Bajenov, Natalia Martinez-Paz, Rupali Jain, John B Lynch, Chloe Bryson-Cahn

**Affiliations:** University of Washington Center for Stewardship in Medicine / Fred Hutchinson Cancer Center, Seattle, Washington; University of Washington, Seattle, Washington; University of Washington Center for Stewardship in Medicine, Salt Lake City, Utah; University of Washington, Seattle, Washington; University of Washington Tele-Antimicrobial Stewardship Program, Seattle, Washington; University of Washington Medical Center-Montlake, Seattle, WA; Harborview Medical Center/University of Washington, Seattle, WA; Harborview Medical Center, Seattle, Washington

## Abstract

**Background:**

Rural and critical access hospitals (CAHs) serve 20% of the United States population. Fewer than 10% of US physicians practice in rural areas and workforce shortages and staff turnover are a constant threat. The University of Washington Center for Stewardship in Medicine (UW CSiM) supports a tele-antimicrobial stewardship (AMS) program (UW TASP ECHO) in small, rural, and CAHs, primarily in the western U.S. with education, mentoring, organizational capacity building, and a community of peers.

**Figure:**
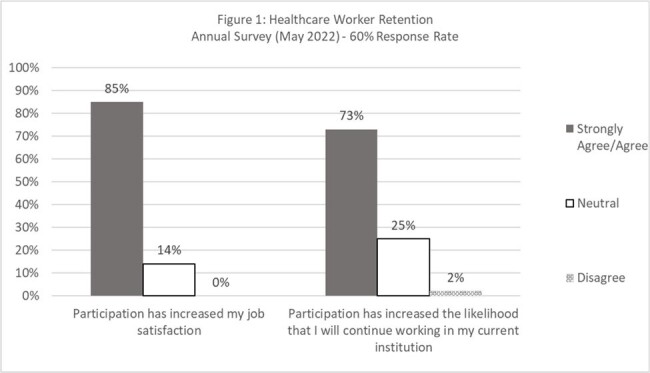

**Methods:**

UW CSiM distributes an annual program evaluation survey to participating hospitals. In 2022, 2 questions were added to the survey and measured with a Likert scale from 1 (strongly disagree) to 5 (strongly agree): 1) Participation in UW-TASP increased my job satisfaction; 2). Participation has increased the likelihood that I will continue working in my institution. Respondents had the option to add free-text comments. Results were summarized descriptively.

**Figure:**
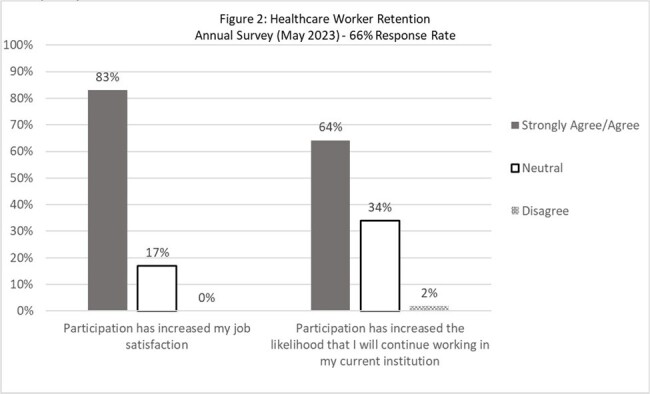

**Results:**

In 2023, 53/80 (66%) of hospitals responded, 44 (83%) met the definition of CAH. Response rate was 60% in 2022. In 2022 and 2023, 85% and 83% agreed or strongly agreed that participation in UW-TASP increased their job satisfaction. Seventy three percent (2022) and 64% (2023) agreed or strongly agreed that participating in UW-TASP increased the likelihood of continuing to work with their current institution. Comments included:

“It's easy to feel "alone" out here in a rural area. Having TASP makes me feel very much a part of a community that I can share my barriers/struggles with and get ideas for how I can do more with my program.”

“Knowledge is power, TASP provides knowledge to make us effective leaders and give the best patient care. That is what gives me job satisfaction, knowing I am helping patients with best practice.”

**Conclusion:**

UW-TASP’s impact expands beyond checking a box that mandates AMS in every acute care hospital. We found establishing and maintaining a relationship with participants allowed us to build a community with and among rural and critical access hospital partners. This study is limited by self-reported survey data but demonstrates that engagement within UW-TASP is associated with increased job satisfaction and rural healthcare worker retention. Further qualitative studies are needed to further explore this.

**Disclosures:**

**All Authors**: No reported disclosures

